# Supplementary figures and images for: Lipocalin 13 enhances insulin secretion but is dispensable for systemic metabolic control
Source: Life Sci Alliance. 2021 Feb 3;4(4):e202000898. doi: 10.26508/lsa.202000898 (PMC7898469; doi:10.26508/lsa.202000898)

Fig S2B

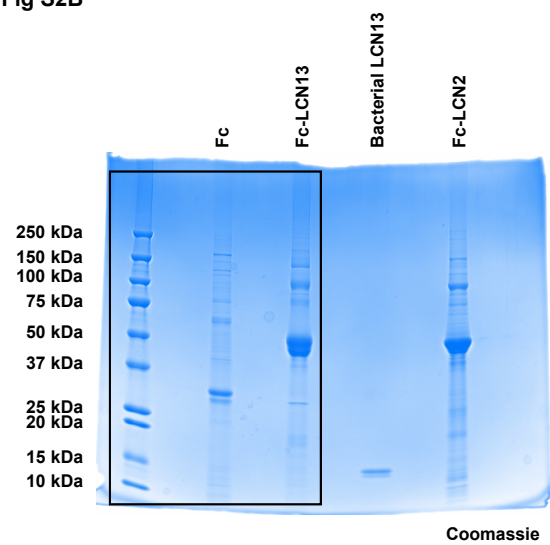

Fig S2C

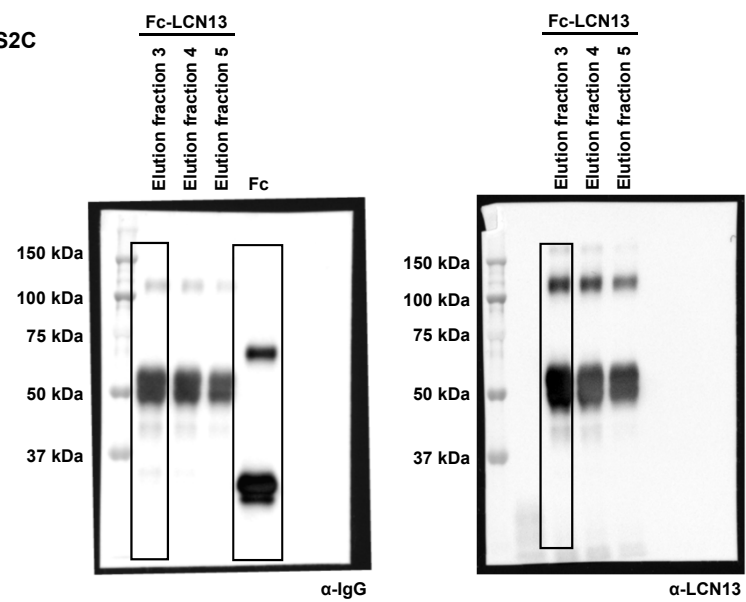

Supplement: Supplementary file 2 [file LSA-2020-00898_SdataFS2.pdf]

Fig 3A

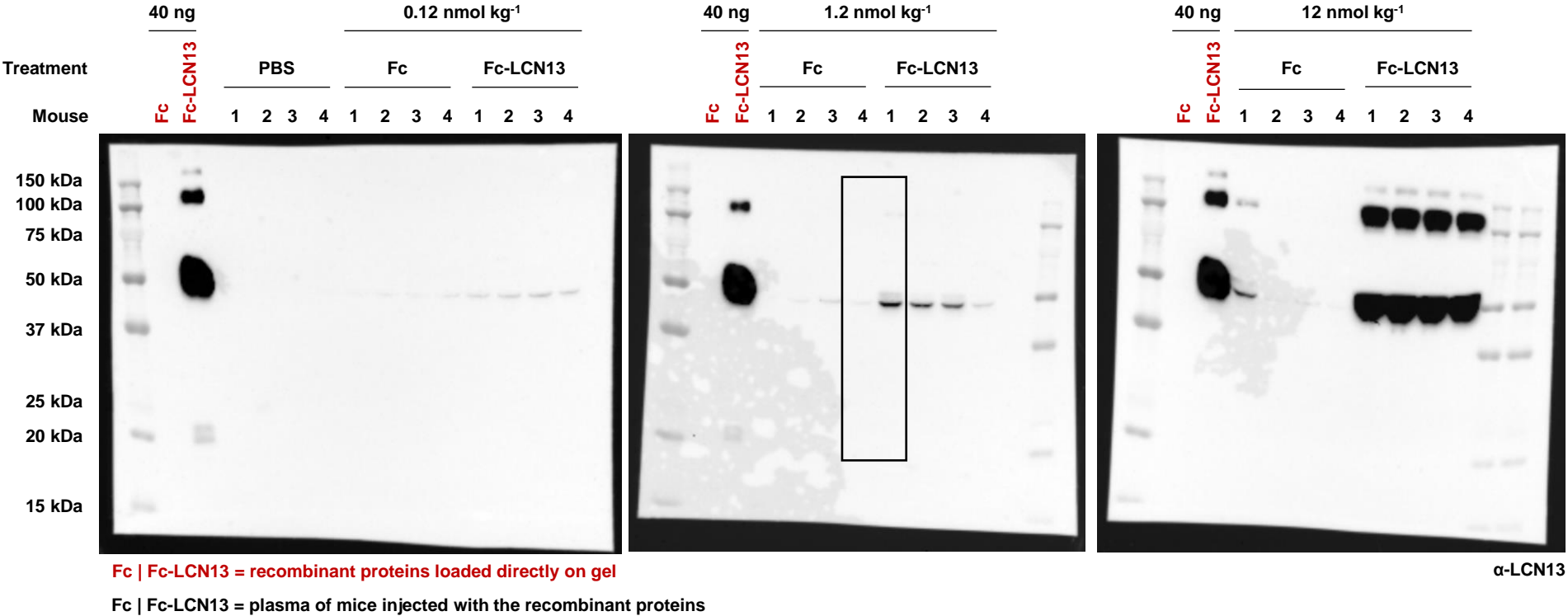

Fig 3F

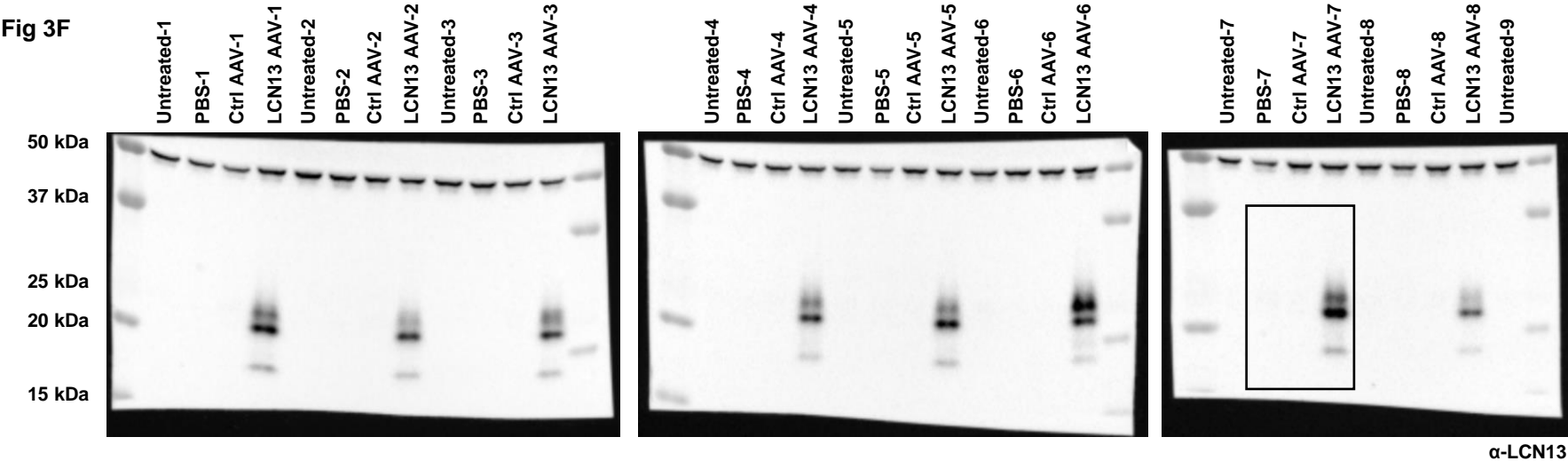

Supplement: Supplementary file 3 [file LSA-2020-00898_SdataF3.pdf]

Fig 4B

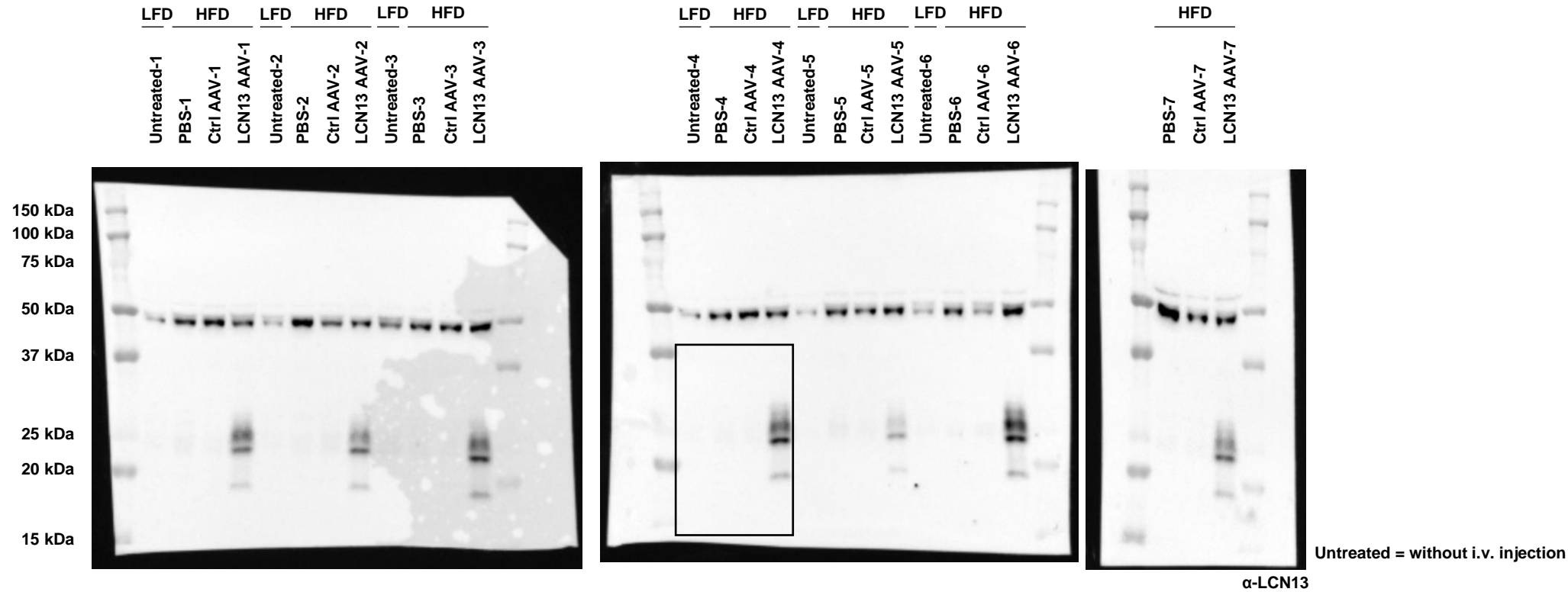

Supplement: Supplementary file 4 [file LSA-2020-00898_SdataF4.pdf]

Fig 6B

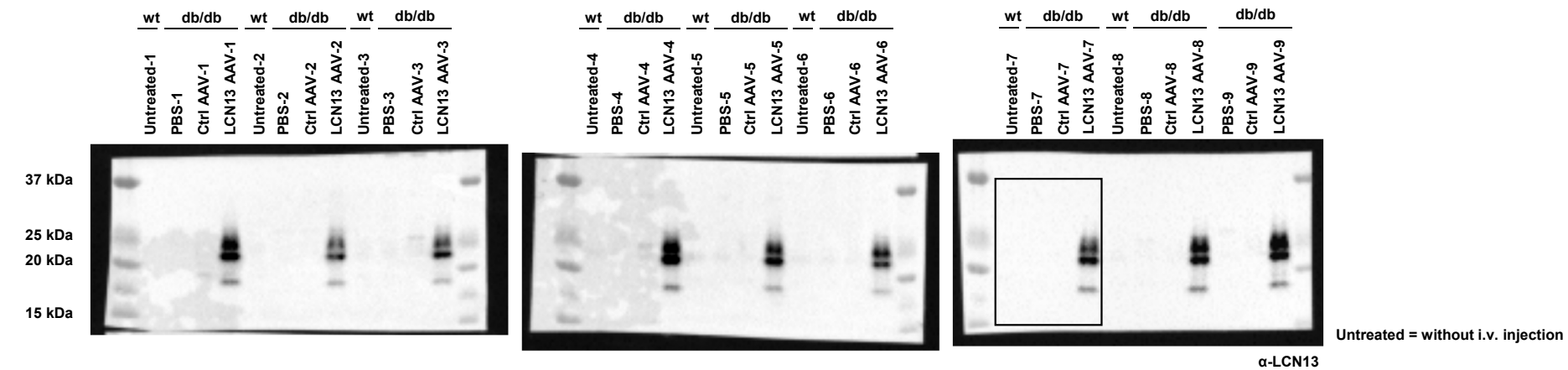

Supplement: Supplementary file 5 [file LSA-2020-00898_SdataF6.pdf]

Fig 7B

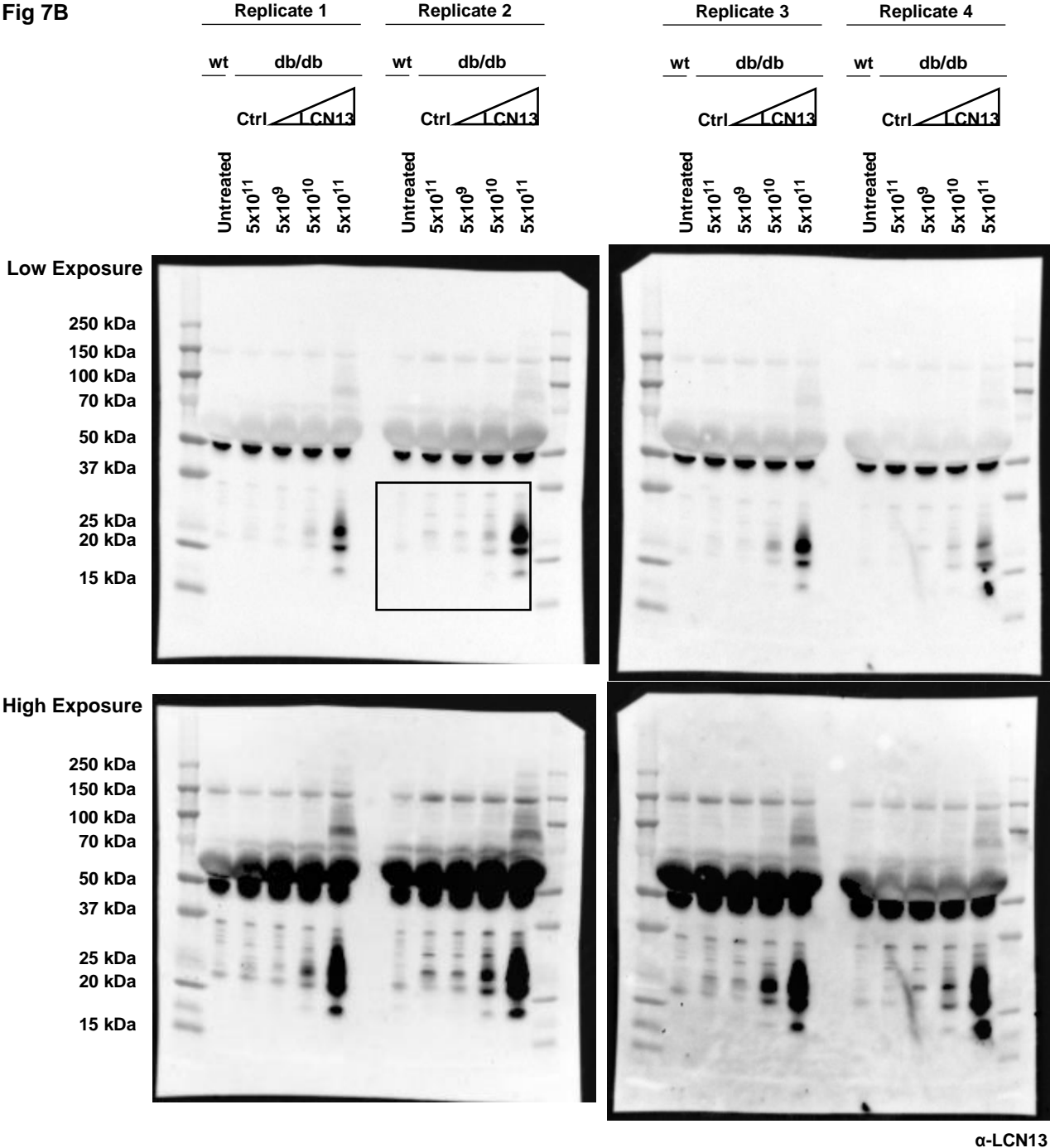

Supplement: Supplementary file 6 [file LSA-2020-00898_SdataF7.pdf]
